# Vascular plants from Pantanal Park Road , Mato Grosso do Sul, Brazil

**DOI:** 10.3897/BDJ.13.e148094

**Published:** 2025-07-03

**Authors:** Marcus V. Santiago Urquiza, Angela Lucia Bagnatori Sartori, Damião Teixeira Azevedo, Daniel de Menezes Mendes, María Silvia Ferrucci, Milena Castello Estra, Ranielly Garcia Silva, Samuel Heimbach Campos, Maria Ana Farinaccio

**Affiliations:** 1 Universidade Federal de Mato Grosso do Sul, Câmpus do Pantanal, Corumbá, Brazil Universidade Federal de Mato Grosso do Sul, Câmpus do Pantanal Corumbá Brazil; 2 Programa de Pós-graduação em Biologia Vegetal, Universidade Federal de Mato Grosso do Sul, Campo Grande, Brazil Programa de Pós-graduação em Biologia Vegetal, Universidade Federal de Mato Grosso do Sul Campo Grande Brazil; 3 Programa de Pós-graduação em Biologia Vegetal, Universidade Estadual de Campinas, Campinas, Brazil Programa de Pós-graduação em Biologia Vegetal, Universidade Estadual de Campinas Campinas Brazil; 4 Instituto de Botánica del Nordeste, Consejo Nacional de Investigaciones Científicas y Técnicas, Universidad Nacional del Nordeste, Corrientes, Argentina Instituto de Botánica del Nordeste, Consejo Nacional de Investigaciones Científicas y Técnicas, Universidad Nacional del Nordeste Corrientes Argentina

**Keywords:** biodiversity, floodplains, inventories

## Abstract

**Background:**

The Pantanal is one of the largest floodplains and the most extensive tropical wetland in the world, covering approximately 139,000 km². It is located in central South America, covering areas in Bolivia, Paraguay and the Brazilian states of Mato Grosso and Mato Grosso do Sul. The Pantanal Park Road (PPR) is an area of special tourist interest located within the Pantanal of Mato Grosso do Sul and covers 6,700 km², passing through the municipalities of Corumbá and Ladário. This data paper plays a crucial role in organising and disseminating essential information on local flora, recognising the value of floristic surveys for understanding regional biodiversity and providing information for conservation efforts. It aimed to provide information to guide public policies for preserving local biodiversity. The floristic survey was generated by combining data from field expeditions with botanical databases, such as JABOT and SpeciesLink. Additional information included life form, substrate type, conservation status and species distribution across Brazilian biomes and states, obtained from Flora e Funga do Brasil using the flora package. Data from Flora e Funga do Brasil were also used to identify new occurrences amongst the listed species.

**New information:**

The vascular plant list of the PPR included 755 species. Most species throughout the PPR were classified as herbs, followed by shrubs or subshrubs, climbers and trees. Thirty species with a known response to fire were identified, of which eight are fire-sensitive, two are fire-tolerant and 21 are fire-stimulated. Of the total species recorded, 710 are native to Brazil, with eight species endemics to Mato Grosso do Sul, ten to the Pantanal and four specifically to the Pantanal in Mato Grosso do Sul. According to the Red List Category from the National Center for Plant Conservation in Brazil, the PPR contains two species classified as Data Deficient (DD), 71 as Least Concern (LC), three as Near Threatened (NT), four as Vulnerable (VU), four as Endangered (EN) and one as Critically Endangered (CR).

## Introduction

The Pantanal is one of the world’s largest floodplains and the most extensive tropical wetland (Damasceno-Junior and Pott 2021). It is located in central South America, covering areas in Bolivia, Paraguay and the Brazilian states of Mato Grosso and Mato Grosso do Sul. It is part of the Upper Paraguay River Basin, covering approximately 139,000 km² (Silva and Abdon 1998), with two-thirds located in Mato Grosso do Sul (Damasceno-Junior and Pott 2021). It borders the Amazon Rainforest to the north and the Cerrado in the central plateau to the east, serving as a crucial centre of biodiversity (Pereira et al. 2012).

Silva and Abdon (1998) divided the Pantanal into 11 subregions based on soil characteristics, flood patterns, topography and vegetation. These subregions are Barão de Melgaço, Cáceres and Poconé in Mato Grosso and Abobral, Aquidauana, Miranda, Nabileque, Nhecolândia, Paiaguás, Paraguai and Porto Murtinho in Mato Grosso do Sul. The annual flood-drought cycles in this region, with distinct rainy and dry seasons, create a diverse environment, varying from highlands that never flood to areas that are permanently submerged (Nunes da Cunha et al. 2022). According to Flora e Funga do Brasil (2024), the Pantanal hosts 1,872 species across 235 plant, fungal and algal families.

The Pantanal Park Road (PPR) is an area of special tourist interest within the Pantanal of Mato Grosso do Sul. It was established by Decree No. 7,122/93, on 17 March 1993 and its routes were initially defined in the late 19^th^ century by Marshal Cândido Rondon, under the names “Estrada Boiadeira” or “Estrada da Manga” (Mato Grosso do Sul 1993). The PPR covers 6,700 km², passing through the municipalities of Corumbá, which encompasses most of its area and Ladário. It starts on highway BR-262 at Buraco das Piranhas and extends for 120 km (Oliveira and Marques 2016). It is crossed by 74 wooden bridges that enable transit over the rivers and wetlands of the Pantanal.

**Figure 1.**
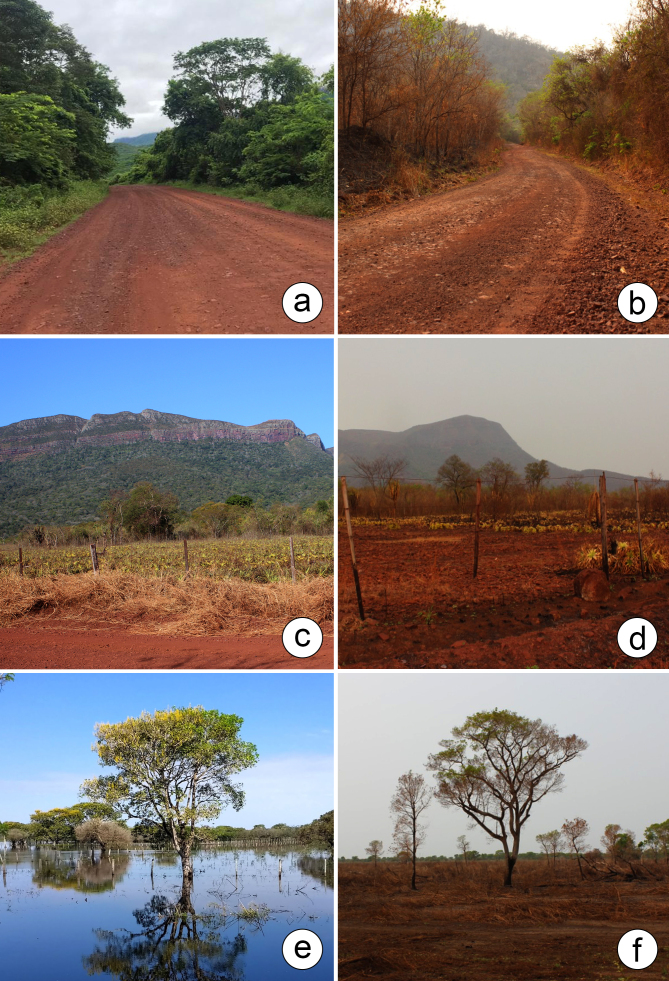
Some physiognomic aspects of the Pantanal Park Road (PPR) in Mato Grosso do Sul, Brazil, comparing the dry and flood periods. **a-b** Semi-deciduous seasonal forest; **c-d** Lateritic bench; **e-f** Alluvial semi-deciduous seasonal forest. Photos by: a, b, d, f. D.M. Mendes; c. M.V.S. Urquiza; e. M.A. Farinaccio.

The PPR represents a nexus of economic, social and environmental factors, including ecotourism, local communities and diverse landscapes, such as hills, forest patches, grasslands, bays and channels (Oliveira and Marques 2016). Some areas within the PPR show signs of anthropogenic alteration and present zones with lateritic benches (laterite crusts), as highlighted by Mendes et al. (2022). In addition, the environments within the PPR have been affected by wildfires in the Pantanal in 2020 (Damasceno-Junior 2021, de Magalhães Neto and Evangelista 2022) and 2022 (César 2022), which have adversely impacted both biodiversity and surrounding communities (Fig. 1).

In this context, analyses to support the development of strategies for restoring and preserving PPR ecosystems are essential, particularly studies focused on its floristic composition. Although significant progress was noted in the botanical knowledge of Pantanal Park Road (e.g. Heimbach Campos and Farinaccio (2021), Mendes et al. (2022), Garcia-Silva et al. (2024), Mendes et al. (2024), Estra (2024)), there is still a lack of robust work that describes the local flora in a satisfactory way.

This data paper plays an important role in organising and disseminating essential information on the local flora, recognising the value of floristic surveys for understanding regional biodiversity and guiding conservation efforts. Given the floristic potential of the area, this data paper presents the outcome of vascular plant surveys conducted throughout the PPR, using data from field collections in the region.

This study aimed to provide valuable information to guide public policies for preserving local biodiversity. By sharing detailed data on the flora of the region, this resource enables the development of effective conservation strategies, sustainable natural resource management and actions to integrate science and environmental governance while engaging the local population (Pereira et al. 2024). Thus, this study contributes directly to ecosystem protection, sustainable regional development and climate change mitigation.

## Sampling methods

### Sampling description

The floristic survey combined data from field expeditions carried out between 2016 and 2024, with botanical databases. Field expeditions were carried out monthly using the walking survey method (Filgueiras et al. 1994). The collected material was herborised according to Fidalgo and Bononi (1989), identified by experts and based on specialised literature and deposited in the Herbarium COR (acronym according to Thiers (2025)).

**Figure 2.**
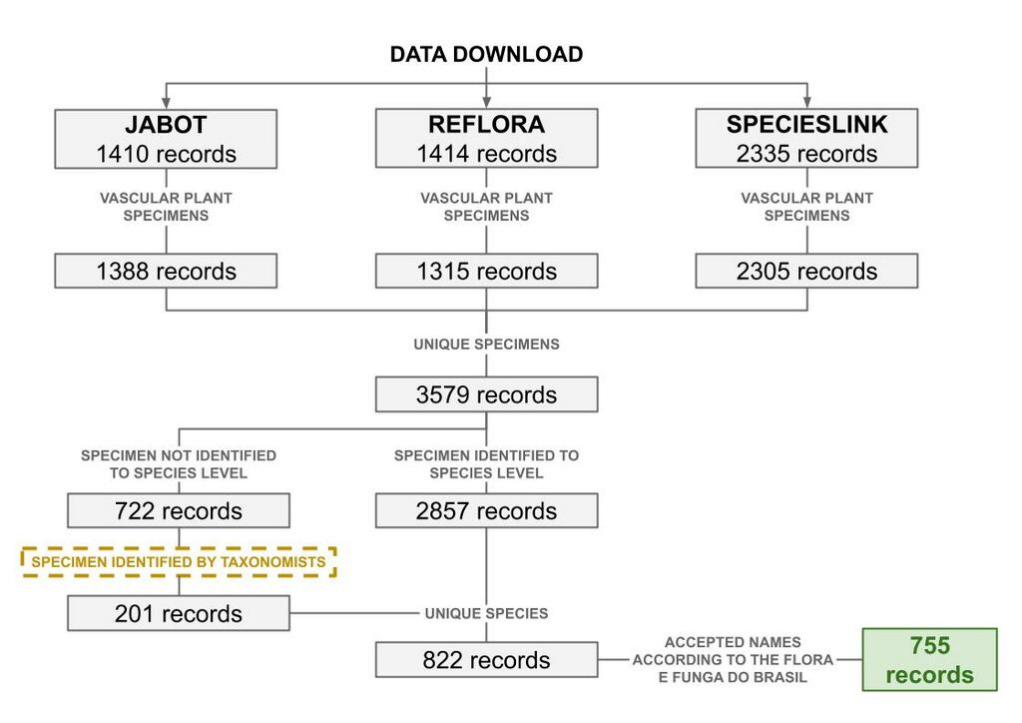
Data processing stages to obtain the species list of vascular plants occurring in the Pantanal Park Road (PPR).

The botanical databases consulted were JABOT (http://www.jbrj.gov.br/jabot), REFLORA (http://reflora.jbrj.gov.br) and SpeciesLink (https://specieslink.net/). Data were collected in May 2024 using the following filters: **Kingdom** = “Plantae”; **Country** = “Brazil”; **State** = “Mato Grosso do Sul”; and **Locality** = “Estrada Parque do Pantanal” & “Estrada Parque Pantanal” & “EPP” & “Porto da Manga” & “Curva do Leque” & “Estrada do Porto da Manga” & “Estrada da Manga” & “Estrada para o Porto da Manga” & “Estrada da Fazenda Bandalta” & “Estrada Boiadeira”. Initially, 5159 specimens were returned (JABOT = 1410, REFLORA = 1414, SpeciesLink = 2335) and subsequently filtered and compiled into a list of 755 species of vascular plants (Fig. 2). The scientific names were compared with the Flora e Funga do Brasil (2024) list and corrected using the flora package (Carvalho 2017) in R 4.4.0 (R Development Core Team 2024). The final list was verified by a group of taxonomists (see Acknowledgements), who also identified the unidentified specimens at the species level. The identified specimens had their registration in the JABOT updated. Infraspecific categories or hybrids were not considered.

**Species traits**: In addition, life form, substrate type and species distribution across Brazilian biomes and states were obtained from Flora e Funga do Brasil (2024) using the flora package (Carvalho 2017). The life forms considered were: a) shrub or subshrub; b) tree; c) herb; and d) climber. The substrate types were classified as: a) aquatic; b) amphibious; c) epiphyte; d) hemi-epiphyte; e) hemi-parasite; f) parasite; g) rupicolous; h) rupicolous and terrestrial; and i) terrestrial. Species classified by Flora e Funga do Brasil (2024) as having both terrestrial and aquatic substrates were considered amphibious. To obtain the conservation status, the Official List of Endangered Species of Brazilian Flora (Brasil 2022) was consulted. Additionally, the species list for the PPR was compared to Damasceno-Junior et al. (2022), which classified several Pantanal species based on their responses to fire. These classifications included fire-sensitive (species that are easily damaged or killed by fire), fire-tolerant (species capable of withstanding certain fire regimes) and fire-stimulated (species that rely on specific fire regimes for the successful development of their life cycle).

**Checking new occurrences**: Data from Flora e Funga do Brasil (2024) were also used to check new occurrences amongst the species on the list. New occurrences were verified for: a) Pantanal; b) Pantanal in Mato Grosso do Sul; c) Mato Grosso do Sul; d) Central-West region; and e) Brazil.

## Geographic coverage

### Description

**Figure 3.**
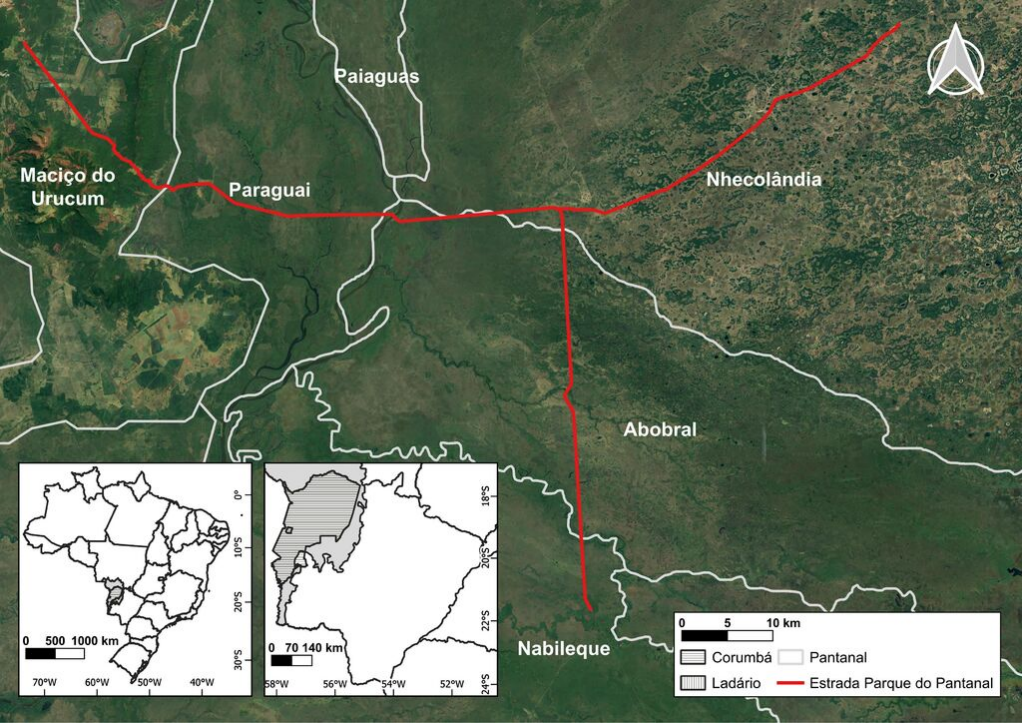
Geographical location of the Pantanal Park Road in Mato Grosso do Sul, Brazil, from Heimbach Campos and Farinaccio (2021).

The PPR is located in the State of Mato Grosso do Sul (Brazil). It starts on highway BR-262 and passes through the subregions Abobral, Nabileque, Nhecolândia and Paraguay (Silva and Abdon 1998), facing the Urucum Massif, which is a mining area (Nunes et al. 2010), totalling 120 km in length, with elevations ranging from 80 to 407 m (Fig. 3). According to the updated Köppen-Geiger Climate Classification (Beck et al. 2018), the climate is tropical with a dry winter season (Aw). It is characterised by average annual temperatures above 18ºC and two well-defined seasons, the rainy season from October to March and the dry season between April and September (Pereira et al. 2012).

**Figure 4.**
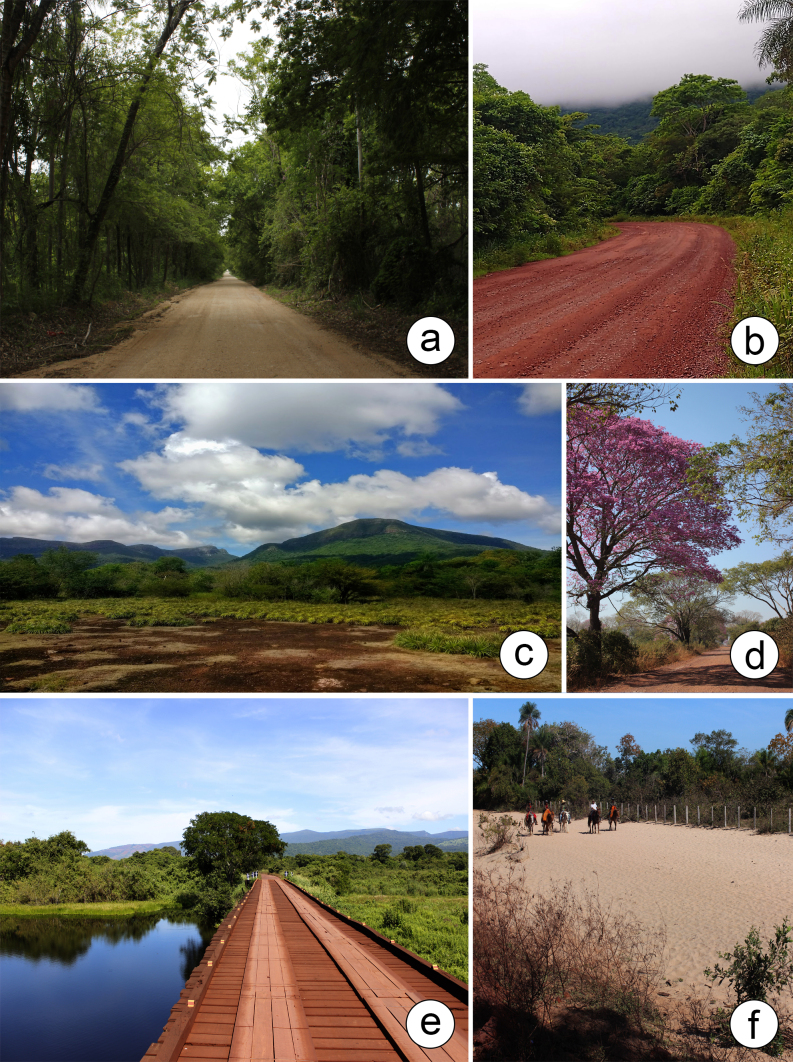
Phytophysiognomies of the Pantanal Park Road in Mato Grosso do Sul, Brazil. **a** Deciduous seasonal forest; **b** Semi-deciduous seasonal forest; **c** Lateritic bench; **d-e** Alluvial semi-deciduous seasonal forest; **f** Cerrado area, close to Savannah. Photos by: a, f. D.M. Mendes; b, c. M.A. Farinaccio; d. R.G. Silva; e. M.V.S. Urquiza.

**Vegetation types**: The vegetation types identified throughout the PPR were classified, based on data compiled by Mendes et al. (2022), namely: Damasceno-Júnior et al. (2009), Negrelle (2013) and Takahasi and Meirelles (2014) (Fig. 4). Areas of a deciduous seasonal forest, semi-deciduous seasonal forest and alluvial semi-deciduous forest were recorded. In addition, species were also observed in relatively anthropogenised environments, which show a high degree of alteration due to human activities, primarily through land use for pasture creation. Another notable finding is the species identified on the lateritic tablelands (cangas) of the Urucum Massif, a specialised habitat characterised by ironstone outcrops. These different environments reflect the ecological complexity of the studied area, highlighting the diversity of habitats that form the vegetation mosaic of the Pantanal, particularly throughout the PPR.

### Coordinates

-57.621 and -56.704 Latitude; -19.069 and -19.089 Longitude.

## Taxonomic coverage

### Description

**Figure 5.**
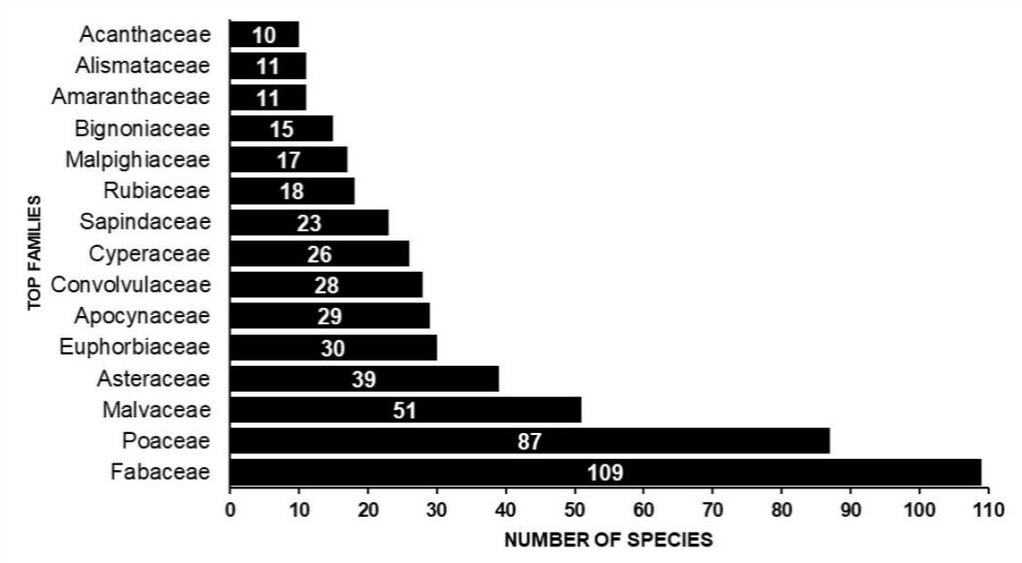
The richest families recorded in Pantanal Park Road. Values inside bars indicate species numbers.

The vascular plant list for the PPR included 755 species (744 angiosperms, 9 ferns and 2 lycophytes) distributed across 413 genera (406 angiosperms, 6 ferns and 1 lycophyte) and 91 families (87 angiosperms, 3 ferns and 1 lycophyte). There are no records of gymnosperms during field expeditions or in databases. The richest families are, respectively, Fabaceae (109 spp., 14.44%), Poaceae (87 spp., 11.52%), Malvaceae (51 spp., 6.75%), Asteraceae (39 spp., 5.17%), Euphorbiaceae (30 spp., 3.97%), Apocynaceae (29 spp., 3.84%), Convolvulaceae (28 spp., 3.71%), Cyperaceae (26 spp., 3.44%), Sapindaceae (23 spp., 3.05%), Rubiaceae (18 spp., 2.38%), Malpighiaceae (17 spp., 2.25%), Bignoniaceae (15 spp., 1.99%), Amaranthaceae (11 spp., 1.46%), Alismataceae (11 spp., 1.46%) and Acanthaceae (10 spp., 1.32%) (Fig. 5). These fifteen families account for 66.76% (i.e. 504 spp.) of the species found in the PPR. This shows that, of the 91 families of vascular plants, only 15 account for two-thirds of all diversity. Of the 91 families, 21 are represented by only one species.

**Figure 6.**
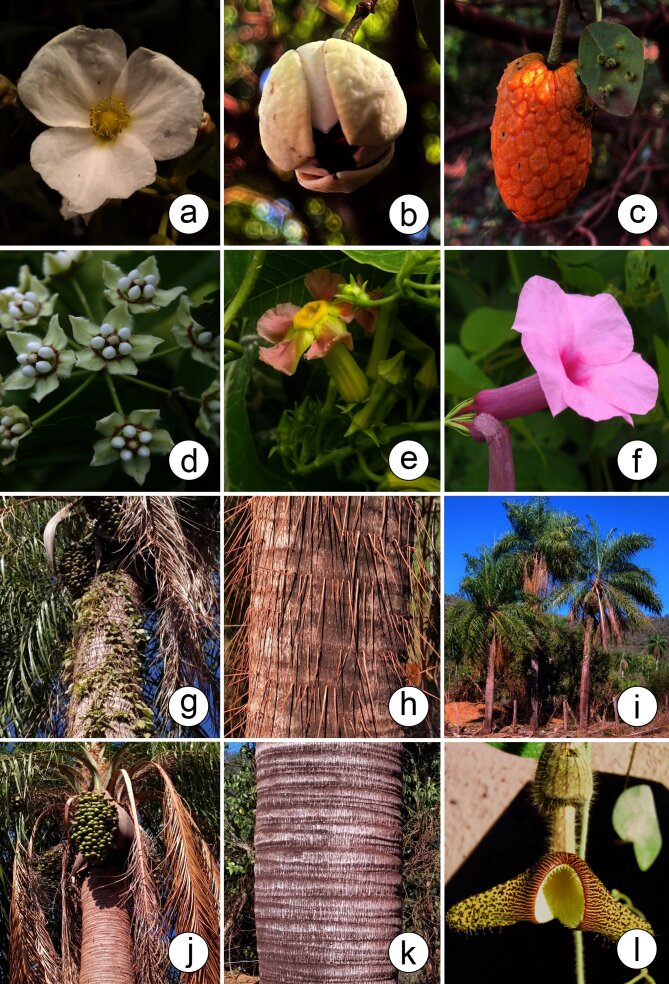
Some species found in the Pantanal Park Road in Mato Grosso do Sul, Brazil. **a** *Echinodoruspaniculatus* Micheli (Alismataceae); **b-c** *Annonanutans* (R.E.Fr.) R.E.Fr. (Annonaceae); **d** *Funastrumclausum* (Jacq.) Schltr. (Apocynaceae); **e** *Prestonialagoensis* (Müll.Arg.) Woodson (Apocynaceae); **f** *Rhabdadeniamadida* (Vell.) Miers (Apocynaceae); **g-h** *Acrocomiaaculeata* (Jacq.) Lodd. ex Mart. (Arecaceae); **i-k** *Acrocomiacorumbaensis* S.A.Vianna (Arecaceae); **l** *Aristolochiaridicula* N.E.Brown (Aristolochiaceae). Photos by: a. R.G. Silva; b-c. D.M. Mendes; d-l. M.A. Farinaccio.

**Figure 7.**
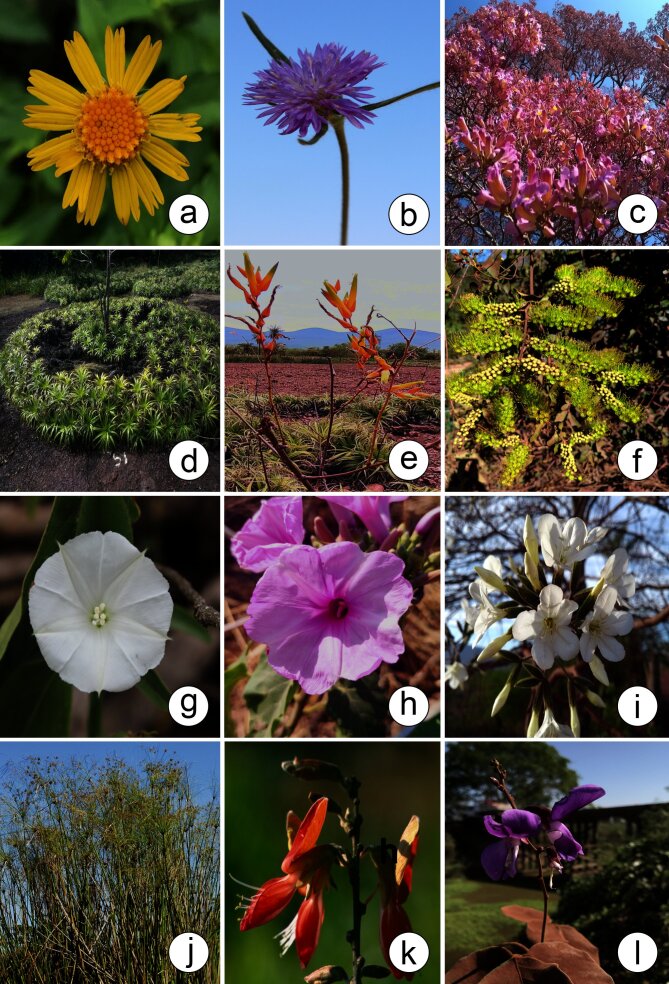
Some species found in the Pantanal Park Road in Mato Grosso do Sul, Brazil. **a** *Calearupicola* Chodat (Asteraceae); **b** *Stilpnopappuspantanalensis* H.Rob. (Asteraceae); **c** *Handroanthusimpetiginosus* (Mart. ex DC.) Mattos (Bignoniaceae); **d-e** *Deuterocohniameziana* Kuntze ex Mez (Bromeliaceae); **f** *Combretumlanceolatum* Pohl ex Eichler (Combretaceae); **g** *Ipomoeaalba* L. (Convolvulaceae); **h** *Ipomoeacarnea* Jacq. (Convolvulaceae); **i** *Cordiaglabrata* (Mart.) A.DC. (Cordiaceae); **j** *Cyperusgiganteus* Vahl (Cyperaceae); **k** *Cerradicolaelliptica* (Desv.) L.P.Queiroz (Fabaceae); **l** *Diocleaburkartii* R.H.Maxwell (Fabaceae). Photos by: a, b, g, l. D.M. Mendes; c, i. M.V.S. Urquiza; d-f, h. M.A. Farinaccio; j, k. P.R. Souza.

**Figure 8.**
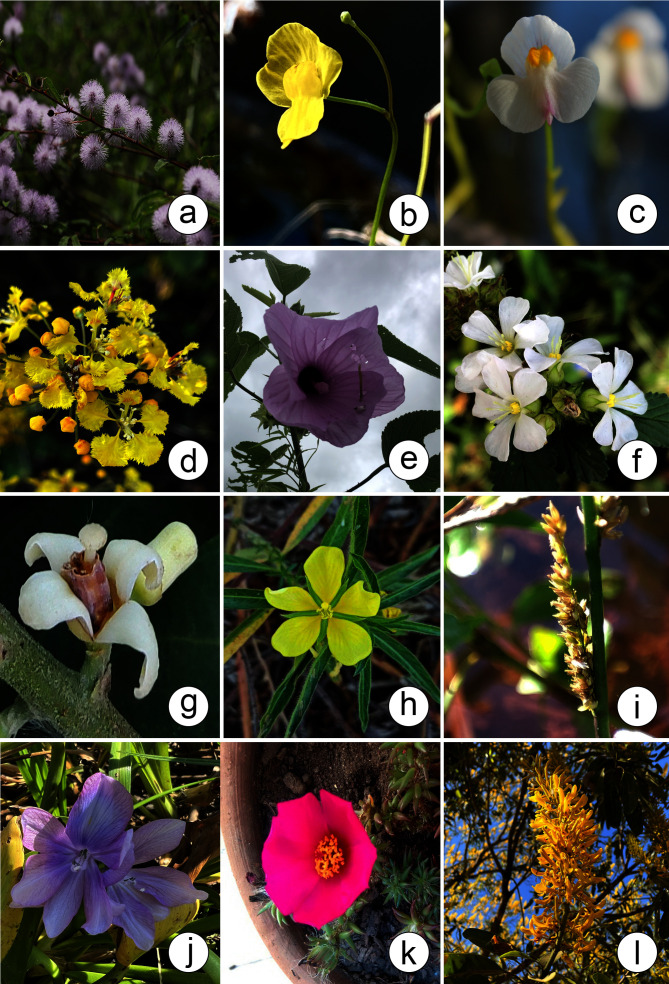
Some species found in the Pantanal Park Road in Mato Grosso do Sul, Brazil. **a** *Mimosapolycarpa* Kunth (Fabaceae); **b** *Utriculariagibba* L. (Lentibulariaceae); **c** *Utriculariapoconensis* Fromm; **d** *Diplopteryslutea* (Griseb.) W.R.Anderson & C.C.Davis (Malpighiaceae); **e** *Hibiscusstriatus* Cav. (Malvaceae); **f** *Melochiaparvifolia* Kunth (Malvaceae); **g** *Guareaguidonia* (L.) Sleumer (Meliaceae); **h** *Ludwigialeptocarpa* (Nutt.) H.Hara (Onagraceae); **i** *Hymenachneamplexicaulis* (Rudge) Nees (Poaceae); **j** *Pontederiacrassipes* Mart. (Pontederiaceae); **k** *Portulacahoehnei* D.Legrand (Portulacaceae); **l** *Vochysiadivergens* Pohl (Vochysiaceae). Photos by: a. P.R. Souza; b, d, l. D.M. Mendes; c, h. R.G. Silva; e, f. M.C. Estra; g, k. M.A. Farinaccio; i. T.R.F. Sinani; j. L.S.S. Messias.

According to Flora e Funga do Brasil (2024), the Pantanal hosts 1,598 species of angiosperms, 47.24% of which are found in the PPR. These species are distributed across 632 genera, of which 65.34% are found in the PPR and 123 families, with 73.98% represented within the PPR. Pott and Pott (2022) documented 2,567 angiosperm species in the Pantanal. Of the 744 angiosperm species listed in this study, 597 had already been listed by Pott and Pott (2022). The prominence of the families Fabaceae (344 species) and Poaceae (302 species) in the Pantanal is similarly reflected in the PPR, which contains 31.68% and 28.80% of species from these families, respectively (Figs 6, 7, 8).

## Traits coverage

### Life forms

Most species in PPR are classified as herbs (265 spp., 35.10%), followed by shrubs or subshrubs (231 spp., 30.60%), climbers (155 spp., 20.53%) and trees (108 spp., 14.31%). The richest families for each life form are as follows: a) herbs: Poaceae (84 spp., 11.13%), Cyperaceae (26 spp., 3.44%), Asteraceae (14 spp., 1.85%), Alismataceae (11 spp., 1.46%) and Fabaceae (9 spp., 1.19%); b) shrubs or subshrubs: Fabaceae (48 spp., 6.36%), Malvaceae (37 spp., 4.90%), Asteraceae (22 spp., 2.91%), Euphorbiaceae (18 spp., 2.38%) and Rubiaceae (11 spp., 1.46%); c) climbers: Convolvulaceae (23 spp., 3.05%), Fabaceae (23 spp., 3.05%), Sapindaceae (22 spp., 2.91%), Apocynaceae (21 spp., 2.78%) and Bignoniaceae (13 spp., 1.72%); d) trees: Fabaceae (29 spp., 3.84%), Malvaceae (10 spp., 1.33%), Moraceae (9 spp., 1.19%), Polygonaceae (5 spp., 0.66%) and Meliaceae (5 spp., 0.66%).

For substrate type, most species are terrestrial (599 spp., 79.34%), followed by aquatic (63 spp., 8.34%), amphibious (53 spp., 4.64%) and rupicolous-terrestrial species (21 spp., 2.78%). Less abundant species were hemi-epiphytes (6 spp., 0.79%), strictly rupicolous (5 spp., 0.66%), epiphytes (4 spp., 0.53%), hemi-parasites (3 spp., 0.40%) and a single parasitic species (1 sp., 0.13%). Fabaceae (106 spp., 14.04%) is the richest family amongst terrestrial species, while Alismataceae (11 spp., 1.46%), Poaceae (17 spp., 2.25%) and Portulacaceae (4 spp., 0.53%) are the richest species amongst aquatic, amphibious and rupicolous-terrestrial species, respectively.

The richness of aquatic and amphibious angiosperms in the PPR reflects the seasonal dynamics typical of the Pantanal, characterised by distinct flood pulses with alternating periods of flooding and drought. Species in this area are adapted to the flood and drought cycles of the Miranda and Paraguay Rivers, which directly influence the ecological conditions of the PPR. Aquatic and amphibious angiosperms in the PPR account for 21.40% of the 524 species recorded in the Pantanal, as reported by Pott and Pott (2022).

### Response to fire

Of the 804 species known to respond to fire as presented in Damasceno-Junior et al. (2022), 274 occur in the PPR, of which 69 are fire-sensitive, 33 are fire-tolerant and 193 are fire-stimulated (complete data in Suppl. material 2). Amongst the fire-sensitive species, 28 herbs, 16 trees, 14 shrubs/subshrubs and 11 climbing plants were listed. As for the fire-tolerant species, 18 trees, eight shrubs/subshrubs, six climbing plants and one herb were listed. Finally, for the fire-stimulated species, 74 shrubs/subshrubs, 61 herbs, 33 climbing plants and 25 trees were counted.

### Distribution and endemism

The PPR contains 710 native species (94.04%) and 45 non-native species (5.96%) in Brazil. Of the total 755 species, 76 (10.07%) are endemic to Brazil, eight (1.06%) are endemic to Mato Grosso do Sul, ten (1.33%) are endemic to the Pantanal and four (0.53%) are endemic to the Pantanal in Mato Grosso do Sul. Table 1 presents the species endemic to the State of Mato Grosso do Sul, the Pantanal and the Pantanal in Mato Grosso do Sul.

According to the data from Flora e Funga do Brasil (2024), five new occurrences were identified for Brazilian flora, specifically: *Anemopaegmaflavum* Morong (Bignoniaceae), *Araujiastuckertiana* (Heger) Fontella & Goyder (Apocynaceae), *Ipomoeacordatotriloba* Dennst. (Convolvulaceae), *I.paludicola* J.R.I.Wood & Scotland and *Gouanialupuloides* (L.) Urb. (Rhamnaceae). Additionally, 41 new occurrences (5.43%) were identified for the Central-West region, 50 (6.62%) for the State of Mato Grosso do Sul, 295 (39.07%) for the Pantanal and five (0.66%) for the Pantanal in Mato Grosso do Sul. Suppl. material 1 lists the taxa that represent new occurrences. Considering the contributions of the new occurrences identified in this study, of the total 755 species that occur in other Brazilian biomes, 451 (59.74%) are found in the Amazon, 410 (54.31%) in the Caatinga, 618 (81.85%) in the Cerrado, 521 (69.01%) in the Atlantic Forest and 170 (22.52%) in the Pampas.

### Conservation status

According to the Official List of Endangered Species of Brazilian Flora (Brasil 2022), the PPR contains three species (0.40%) classified as Vulnerable (VU), five (0.66%) as Endangered (EN) and one (0.13%) as Critically Endangered (CR). Table 2 presents the species classified as VU, EN or CR.

## Usage licence

### Usage licence

Creative Commons Public Domain Waiver (CC-Zero)

## Data resources

### Data package title

Floristic survey of vascular plants from the Pantanal Park Road in Mato Grosso do Sul (Brazil)

### Resource link

https://doi.org/10.5281/zenodo.14751853

### Number of data sets

1

### Data set 1.

#### Data set name

pantanal-park-road-survey-v4.txt

#### Data format

txt

#### Download URL

https://zenodo.org/records/14751853/files/pantanal-park-road-survey-v4.txt?download=1

#### Description

Dataset containing information on vascular plant species from the Pantanal Park Road in Mato Grosso do Sul (Brazil). It contains 755 vascular plant species with taxonomic and voucher information for each species.

**Data set 1.** 

Column label	Column description
clade	Taxonomic group.
family	Botanical family names.
genus	Botanical genus names.
specificEpithet	Botanical species epithet names.
scientificNameAuthorship	Species authors’ names.
scientificName	Taxon based on Flora e Funga do Brasil (2024).
collectionCode	Acronym of the collection.
collectionBarcode	Barcode of the specimen in the collection.
recordedBy	Name of the specimen collector.
recordNumber	Collector number.
databaseOriginName	Database of origin.

## Additional information

This study highlights the PPR as a significant representation of the southern Pantanal. Covering four of the 11 subregions of the biome, the PPR has a rich diversity of flora and hosts nearly half of the species known in the Pantanal. In addition, the dominant plant families in the biome, Fabaceae and Poaceae, are also prominent in the PPR. These findings can guide public policies focused on preserving the biodiversity of the PPR, supporting both conservation and sustainable management strategies for natural resources. Our research includes a detailed species list, categorised by fire response, endemism in the State of Mato Grosso do Sul and conservation status in the Pantanal and southern Pantanal regions. This approach strengthens the integration of science and environmental management and directly contributes to ecosystem protection, sustainable regional development and climate change mitigation.

## Supplementary Material

18A44DF8-D767-5199-A010-B486C6C44287 10.3897/BDJ.13.e148094.suppl1 Supplementary material 1 New species records Data type taxonomic Brief description List of new species records for Pantanal, Pantanal in Mato Grosso do Sul, Mato Grosso do Sul, Central-West region and Brazil, according to Flora e Funga do Brasil (2024). File: oo_1233577.csv https://binary.pensoft.net/file/1233577 Urquiza et al. (2025)

75B95BCC-FED0-533F-9ED5-73B406CD3756 10.3897/BDJ.13.e148094.suppl2 Supplementary material 2 Species from the Pantanal Park Road and fire responses Data type taxonomic Brief description List of species from Pantanal Park Road, life forms and fire responses according to Damasceno-Júnior et al. (2022). File: oo_1296252.csv https://binary.pensoft.net/file/1296252 Urquiza et al. (2025)

## Figures and Tables

**Table 1.** Species from the Pantanal Park Road endemic to the State of Mato Grosso do Sul, the Pantanal and the Pantanal in Mato Grosso do Sul.

**Family**	**Species**	**Endemism**		
		* **MS**	* **P**	* **P-MS**
Amaranthaceae	*Gomphrenacentrota* E.Holzh.	-	X	-
Amaryllidaceae	*Habranthuspantanalensis* Ravenna	-	X	-
	*Zephyranthespantanalensis* (Ravenna) R.S.Oliveira & Dutilh	X	-	-
Asteraceae	*Lepidaploaamambaia* H.Rob.	X	-	-
	*Stilpnopappuspantanalensis* H.Rob.	-	X	-
Cleomaceae	*Tarenayaeosina* (J.F.Macbr.) Soares Neto & Roalson	X	-	-
Euphorbiaceae	*Jatrophaweddeliana* Baill.	-	X	-
Fabaceae	*Arachisappressipila* Krapov. & W.C.Greg.	-	-	X
	*A.hoehnei* Krapov. & W.C.Greg.	-	-	X
	*A.valida* Krapov. & W.C.Greg.	-	-	X
Malpighiaceae	*Thryallislaburnum* S.Moore	X	-	-
Malvaceae	*Pavonialaetevirens* R.E.Fr.	-	X	-
Moraceae	*Ficuscarautana* L.J.Neves & Emygdio	-	X	-
Portulacaeae	*Portulacahoehnei* D.Legrand	-	-	X

*MS: Mato Grosso do Sul; P: Pantanal; P-MS: Pantanal in Mato Grosso do Sul.

**Table 2.** Threatened species from the Pantanal Park Road according to the Official List of Endangered Species of Brazilian Flora.

**Family**	**Species**	**Red List Category**
Amaranthaceae	*Gomphrenacentrota* E.Holzh.	EN
Apocynaceae	*Aspidospermaparvifolium* A.DC.	EN
Cactaceae	*Echinopsiscalochlora* K.Schum.	CR
Convolvulaceae	*Evolvuluschrysotrichos* Meisn.	EN
	*Ipomoeasubrevoluta* Choisy	VU
Malpighiaceae	*Thryallislaburnum* S.Moore	VU
Moraceae	*Ficuscarautana* L.J.Neves & Emygdio	EN
Olacaceae	*Dulaciaegleri* (Bastos) Sleumer	EN
Plantaginaceae	*Stemodiahyptoides* Cham. & Schltdl.	VU
